# Effects of curcumin on cardiovascular risk factors in obese and overweight adolescent girls: a randomized clinical trial

**DOI:** 10.1590/1516-3180.2018.0454120419

**Published:** 2019-11-04

**Authors:** Sahar Saraf-Bank, Alireza Ahmadi, Zamzam Paknahad, Mohammadreza Maracy, Mojgan Nourian

**Affiliations:** I MSc. Doctoral Student and Dietitian, Students’ Research Committee, Isfahan University of Medical Sciences; Doctoral Student and Dietitian, Food Security Research Center, Isfahan University of Medical Sciences; and Doctoral Student and Dietitian, Department of Community Nutrition, School of Nutrition and Food Science, Isfahan University of Medical Sciences, Isfahan, Iran.; II MD. Associate Professor and Cardiologist, Pediatric Cardiovascular Research Center, Cardiovascular Research Institute, Isfahan University of Medical Sciences, Isfahan, Iran.; III PhD. Full Professor and Dietitian, Food Security Research Center, Isfahan University of Medical Sciences, and Full Professor and Dietitian, Department of Clinical Nutrition, School of Nutrition and Food Science, Isfahan University of Medical Sciences, Isfahan, Iran.; IV PhD. Full Professor and Statistician, Department of Epidemiology and Biostatistics, School of Health, Isfahan University of Medical Sciences, Isfahan, Iran.; V PhD. Assistant Professor and Dietitian, Food Security Research Center, Isfahan University of Medical Sciences, and Assistant Professor and Dietitian, Department of Community Nutrition, School of Nutrition and Food Science, Isfahan University of Medical Sciences, Isfahan, Iran.

**Keywords:** Curcumin, Adolescent, Diet, reducing, Metabolic syndrome

## Abstract

**BACKGROUND:**

Obese adolescents are at higher risk of development of cardiovascular risk factors and obesity in later life. Dietary intake of antioxidants, particularly curcumin, as an active ingredient of turmeric extract, may have noticeable effects on obesity and its important complications such as cardiovascular risk factors. Therefore, the aim of this study was to assess the effects of curcumin supplementation on cardiovascular risk factors among overweight and obese female adolescents.

**DESIGN AND SETTING:**

Randomized placebo-controlled clinical trial; Pediatric Cardiovascular Research Center, Isfahan, Iran.

**METHODS:**

60 adolescent girls (aged 13-18 years) were randomly assigned to receive either placebo or intervention. The adolescents were asked to consume one 500 mg tablet per day, containing either standardized 95% turmeric extract or placebo, and to undergo a weight maintenance or a mild weight loss diet for 10 weeks. Anthropometric and biochemical indices were assessed at the baseline and the end of the intervention.

**RESULTS:**

Curcumin supplementation had beneficial effects on body mass index (P = 0.019), waist circumference (P = 0.008), hip circumference (P = 0.030), high-density lipoprotein levels (P = 0.042) and triglyceride/high-density lipoprotein ratio (P = 0.021). However, in univariate analysis of covariance, no significant differences were found between the intervention and placebo groups after 10 weeks of supplementation (P > 0.05).

**CONCLUSIONS:**

Prescription of curcumin supplementation along with use of a slight weight loss diet might have beneficial effects on some cardiovascular risk factors among overweight and obese female adolescents. Larger clinical trials with higher curcumin doses and longer duration are needed to confirm the results from the current study.

## INTRODUCTION

Today, childhood obesity is becoming a serious problem worldwide.^1^ It has been shown that overweight adolescents are at greater risk of comorbidities relating to obesity in adulthood, like ischemic heart disease.^2^ The prevalence of overweight and obesity have been rapidly increasing in developing countries. These are around 5%-13.5% and 3.2%-11.9%, respectively, among Iranian adolescents.^3^ It seems that childhood obesity (i.e. at ages less than 18 years) may be an important predictor for comorbidities in adulthood, such as hypertension and dyslipidemia.^4^ Therefore, finding a practical solution for this problem is of utmost importance.

It has been reported that several lifestyle factors, including duration of nocturnal sleeping, practicing of physical activity and the length of time spent watching television can be classified as possible causes of childhood obesity.^1^ Moreover, children’s dietary intake is a noticeable factor relating to development of central and general obesity among children and adolescents.^5^^,^^6^

One study has shown that some types of foods, like spices, may play an important role in combating obesity and its associated complications.^7^ In this context, interest has increasingly been focused on turmeric extract, given that curcumin is its active ingredient. Several studies have reported on the possible effects of curcumin against obesity, inflammation, diabetes and cardiovascular disease and related risk factors.^7^^,^^8^^,^^9^ However, the results from the available studies assessing the effect of curcumin on lipid profiles have been contradictory.^10^^,^^11^ One meta-analysis showed that curcumin supplementation did not have any beneficial effect on lipid fractions.^9^ In addition, the existing results regarding glycemic indices have been insufficient and inconsistent.^11^^,^^12^

Based on our review of the literature, we did not find any study assessing the potential effects of curcumin supplementation on cardiovascular risk factors among adolescents. Therefore, selection of this target group to determine the possible effects of curcumin is of great importance.

## OBJECTIVE

The aim of this study was to assess the effects of curcumin supplementation on cardiovascular risk factors, including anthropometric measurements, glycemic indices and lipid profile among overweight and obese female adolescents in Iran.

## METHODS

### Study design and setting

This was a randomized parallel clinical trial conducted at the Pediatric Cardiovascular Research Center, Isfahan University of Medical Sciences, Isfahan, Iran. The present clinical trial report followed the CONSORT checklist.

### Ethical issues

This study was approved by the ethics committee of Isfahan University of Medical Sciences, Isfahan, Iran (no. 396160; July 2017). The registration number of this study in the Iranian Registry of Clinical Trials (http://www.irct.ir/) is IRCT20171107037302N1. Before the start of the study, oral and written informed consents were obtained from the adolescents and from one of their parents, respectively.

### Participants

Overweight or obese female adolescents were recruited for this trial, which was conducted over a 10-week period. These adolescent girls (aged between 13-18 years) were included in this randomized placebo-controlled clinical trial between September 2017 and January 2018, at the Pediatric Cardiovascular Research Center, Isfahan, Iran. This research center has a clinic for overweight and obese children and adolescents aged 6-18 years, and the medical records available at this center go back as far as 1992. From all over the city, general practitioners and some relevant specialists such as endocrinologists refer overweight and obese children to this center to be enrolled in its weight management program. At this clinic, children are encouraged to increase their physical activity, reduce their empty calorie intake (calories from solid fats and added sugar), select healthier foods and improve their lifestyle (through measures such as reducing their television time).

From 1,050 medical records that were screened by the present researchers, 227 adolescents were deemed to be potentially eligible participants (for being overweight or obese). Out of these 227 potentially eligible adolescents, 105 of them were not available, 27 were unwilling to participate in the study, 17 were suffering from chronic diseases or were consuming drugs, 14 were on a specific diet in other weight management clinics and 4 had not yet started to have a menstrual cycle. In total, 60 post-pubescent overweight or obese adolescent girls were included in accordance with the inclusion criteria described below. Body mass index percentile for age between the 85^th^ and 95^th^ was considered to represent overweight and more than the 95^th^ was considered to represent obesity, in accordance with the World Health Organization criteria.^13^

Participants were selected based on the following inclusion criteria: having had a menstruation cycle for more than six months; absence of any history of chronic diseases (diabetes, hypo and hyperthyroidism, liver, renal and cardiovascular disease and polycystic ovary syndrome); absence of medication use (drugs affecting the metabolism of lipids and carbohydrates, such as statins, metformin, hormone therapy drugs, multivitamin minerals, corticosteroids and non-steroidal anti-inflammatory drugs); and absence of any history of weight reduction over the past three months. Participants would be excluded if they had poor compliance with the diet and supplements that were administered (use of less than 80% of the supplements) and if they were not willing to continue in the study.

### Study conduction

#### Random sequence generation and allocation concealment

Random permuted blocks (dual blocks) of participants based on BMI percentile (85^th^ < BMI percentile < 95^th^; and BMI percentile ≥ 95^th^), according to the data obtained from the files available in the pediatric cardiovascular research center were formed by the researchers. A trained employee of the pediatric cardiovascular research center who was blinded to the random allocation sequence assigned adolescents to the curcumin and placebo groups using SPSS software-generated random numbers.

#### Blinding procedures

To maintain blinding, the curcumin supplement and its quite similar placebo (shape, color, odor and label) were produced and encoded as A and B by Karen Critical Pharmaceutical and Nutritional Supplements Company, Tehran, Iran. Thereafter, supplements A and B were randomly assigned to each arm of the study. Accordingly, the researcher, participants and statistics analyst were completely unaware of the assigned intervention.

### Intervention

A weight maintenance diet was prescribed for those with BMI between the 85^th^ and 95^th^ percentile and a weight reduction diet (maximum of one pound, or 454 grams, per month) for those with BMI more than the 95^th^ percentile.^16^ The prescribed diet was designed based on healthy eating advice.^17^ The distribution of macronutrients in the prescribed diets was 50%-60% for carbohydrates, 15%-20% for protein and 30% for total fat, which is similar to the regular Iranian pattern.^18^ The participants received the precise amount of each food group along with a sample food menu. In addition, a comprehensive exchange list was given to these adolescents. Before the start of the study, group sessions were held for the adolescents and their mothers. In these sessions, the proper way of using the sample food menu and exchange list was taught to both the adolescents and their mothers.

The adolescents were asked to consume one 500 mg tablet per day, containing either standardized 95% turmeric extract or placebo, with their meal for 10 weeks. During the study period (at week 5), the participants completed a physical activity questionnaire and a three-day dietary record (one weekend day and two weekdays).

At the end of the study period, fasting blood samples were collected. In addition, anthropometric indices and the blood pressure of all subjects were measured. The physical activity questionnaire and three-day dietary records were completed by all participants.

To check the participants’ compliance with curcumin supplement use, phone calls were made to both the adolescents and their mothers every fortnight. Furthermore, the adolescents were asked to complete a checklist and to return the supplement containers at the end of week 10. The adolescents’ adherence to the diet was checked from the dietary records that they completed during the study.

### Outcomes

We made preliminary and final assessments of the outcomes. The primary outcomes were anthropometric measurements and glycemic indices and the secondary outcome was the lipid profile.

At the beginning of the study, fasting blood samples were collected and the anthropometric indices and blood pressure of the adolescents were measured. General information was obtained from all the participants, including the parents’ education level and occupation, family income, smoking history, past medical history and medication use. Furthermore, a validated physical activity questionnaire^14^ and a three-day dietary record (one weekend day and two weekdays) were completed by the adolescents. Total energy expenditure for each adolescent was calculated in accordance with the recommendations from the Institute of Medicine.^15^

### Daily dietary intakes and physical activity assessment

Dietary intakes were assessed using a three-day dietary record (one weekend day and two weekdays) at the beginning (week 1), middle (week 5) and end (week 10) of the study period. All participants were instructed on how to record their dietary intakes before the intervention started. After the adolescents had completed the dietary records, the precise weight in grams of each food item was determined by the researchers through household measurements. The daily dietary intake of each food item was entered into the Nutritionist IV software (First Databank Division, The Hearst Corporation, San Bruno, CA, USA) for analysis on total energy intake and macro and micronutrients.

The adolescents’ physical activity levels were assessed through a validated physical activity questionnaire^14^ at baseline and at weeks 5 and 10. This questionnaire included nine physical activity levels that were graded based on their respective metabolic equivalent of task (MET).

### Blood pressure and anthropometric measurements

Anthropometric indices were measured at baseline and at the end of the study period. Weight was measured using a Seca scale, to the nearest 0.1 kg, with the subjects barefoot and wearing light clothing. Height was measured using a wall-mounted tape measure, to the nearest 0.5 cm, without shoes. Body mass index was calculated by dividing weight (kg) by height squared (m^2^). Waist circumference and hip circumference were measured using an inelastic tape with an accuracy of 0.1 cm, at the midpoint between the lower rib and top of the iliac crest and at the largest hip circumference, respectively.

After 10 minutes of resting in a sitting position, blood pressure was measured twice using a digital sphygmomanometer (OMRON, M3, HEM-7134-E), and the average of these two measurements was used for the statistical analysis.

### Biochemical assessment

Blood samples of 10 ml were taken after overnight fasting (12-14 hours), at baseline and after 10 weeks of the intervention. These were centrifuged at 3000 rpm for 15 minutes and were stored at -70 °C until use. The lipid profile, including total cholesterol, low-density lipoprotein, high-density lipoprotein and triglycerides, was assessed using commercially available enzyme assay kits (Pars Azmoon, Tehran, Iran). Colorimetric assay kits were used to determine fasting blood sugar (Pars Azmoon, Tehran, Iran). Insulin levels were measured by means of the enzyme-linked immunosorbent assay (ELISA) method (Hangzhou Eastbiopharm Co. Ltd., Hangzhou, China). The inter and intra-assay variations of the insulin assessment were less than 10%. The homeostasis model assessment for insulin resistance was calculated based on the following formula: [Fasting plasma insulin (U/ml) × Fasting blood glucose (mg/dl)]/405.^19^

### Statistical analysis

The sample size was calculated based on a formula that is available for parallel trials.^20^ The type 1 error (α) was considered to be 0.05; the type 2 error (β) was 0.1 (i.e. power = 90); d (the significant difference in fasting plasma glucose levels) was 0.38; S_1_ (the standard deviation of the fasting plasma glucose level in the control group) was 0.32; and S_2_ (the standard deviation of the fasting plasma glucose level in the intervention group) was 0.45.^21^ Based on these data, 22 participants were required for each arm of the study. To account for possible withdrawals during the study, we included 30 adolescents in each group, i.e. in the intervention and placebo groups.

Presence of normal distribution of variables was assessed using Q-Q plots and the Shapiro-Wilk test. Logarithmic transferred means were used for variables with an unusual distribution. Linear regression was applied to predict the approximate values of missing data. In the current study, there were only five withdrawals (less than 10% of the study population). In addition, there was a high correlation between the baseline and endpoint values. Therefore, linear regression had the capacity to predict missing data with the right precision. In this method, endpoint values of variables were considered to be dependent variables and baseline values were considered to be independent variables. In the output from the linear regression analysis, there were constant values in the coefficient table. Through using the formula “y (endpoint) = x (baseline)+b (constant value)”, the endpoint values of the variables could be predicted.

The general features of the participants were compared by means of the independent-sample t test and the chi-square test. In addition, dietary intakes, physical activity levels and between-group comparisons were checked using an independent-sample t test. For within-group analyses, a paired-sample t test was applied. Univariate analysis of covariance (ANCOVA) was used to compare between-group differences. Confounding factors were detected by conducting a correlation test between potential confounding factors and the baseline values of the variables. Variables with r correlation ≥ 0.2 were considered to be confounding factor. In this regard, the potential effects of physical activity level, turmeric powder intake and intakes of some dietary antioxidants such as vitamin C, vitamin E, selenium and beta-carotene were controlled for, in the adjusted model.

All variables were reported as the mean ± standard deviation (SD). The Statistical Package for the Social Sciences, version 18, was used for the statistical analyses (SPSS Inc., Chicago, IL, USA). P-values less than 0.05 were considered to be significant in this study.

## RESULTS

**Figure 1.**
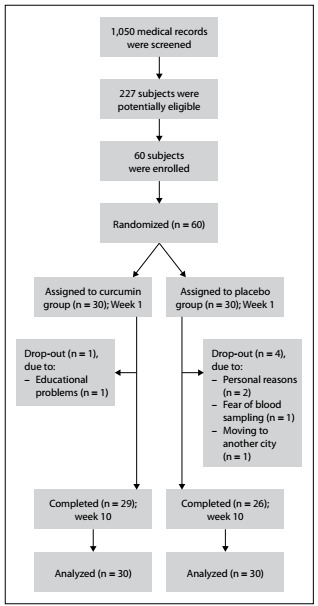
Flowchart of the study.

Over the course of the study period, five adolescents withdrew from the study based on personal reasons, educational problems including conflicting school appointments, fear of blood sampling and moving to another city (Figure 1). Nevertheless, through prediction of the missing values, the data of all 60 participants were entered into the statistical analysis (30 in each group).

There were no substantial side effects among the participants. However, some adolescents had mild headaches and nausea, which were resolved through continual use of curcumin capsules.

Approximately seven months were spent on sampling, subject enrollment, completion of the intervention and data collection. The baseline characteristics of the participants are presented in Table 1. As shown in this table, there were no significant baseline differences in age, overweight/obesity distribution or socioeconomic status among the participants.

**Table 1.** General baseline characteristics of the female adolescents

Variables	Curcumin (n = 30)	Placebo (n = 30)	P-value^1^
Age (years); mean ± standard deviation	16.03 ± 1.56	15.98 ± 1.72	0.907
Overweight/obese status; n (%)			
Overweight	9 (30.0)	11 (36.7)	0.584
Obese	21 (70.0)	19 (63.3)	
Father’s education level; n (%)			
Illiterate	1 (3.3)	0 (0.0)	0.493
< 12 years	10 (33.3)	13 (43.3)	
12 years	13 (43.3)	9 (30.0)	
> 12 years	6 (20.0)	8 (26.7)	
Mother’s education level; n (%)			
Illiterate	0 (0.0)	0 (0.0)	0.955
< 12 years	9 (30.0)	8 (26.7)	
12 years	15 (50.0)	16 (53.3)	
> 12 years	6 (20.0)	6 (20.0)	
Father’s job; n (%)			
Employee at non-executive level	7 (23.3)	12 (40.0)	0.345
Self-employed	21 (70.0)	15 (50.0)	
Worker at executive level	2 (6.7)	2 (6.7)	
Dead	0 (0.0)	1 (3.3)	
Mother’s job; n (%)			
Employee	3 (10.0)	3 (10.0)	1.000
Self-employed	1 (3.3)	1 (3.3)	
Housewife	26 (86.7)	26 (86.7)	
Family income; n (%)			
Low	2 (6.7)	6 (20.0)	0.252
Moderate	24 (80.0)	22 (73.3)	
high	4 (13.3)	2 (6.7)	
Smoking in family, n (yes%)	5 (16.7)	5 (16.7)	1.000

^1^P-values are from the chi-square test and the independent-sample t test.

Table 2 shows the participants’ daily dietary intakes during the study period. There were no substantial differences in total energy or micro and macronutrients between the two groups. In addition, no significant difference in the physical activity levels of the participants was detected between the two groups (34.70 ± 6.40 MET-hour/day in the curcumin group and 36.82 ± 8.00 MET-hour/day in the placebo group; P-value = 0.262).

**Table 2.** Daily dietary intakes of the female adolescents during the study^1^

Variables	Curcumin (n = 30)	Placebo (n = 30)	P-value^2^
Energy (kcal)	1930.93 ± 365.16	1832.67 ± 330.20	0.279
Carbohydrate (g)	285.24 ± 58.78	271.52 ± 59.83	0.374
Protein (g)	54.34 ± 1.20	57.79 ± 1.25	0.199
Fat (g)	66.95 ± 21.96	61.38 ± 20.73	0.317
Saturated fat (g)	18.82 ± 1.54	17.03 ± 1.56	0.358
Polyunsaturated fat (g)	16.16 ± 5.42	15.15 ± 5.25	0.465
Monounsaturated fat (g)	21.83 ± 9.38	19.66 ± 8.59	0.354
Zinc (mg)	6.73 ± 1.28	6.77 ± 1.26	0.968
Potassium (mg)	2739.27 ± 638.48	2516.01 ± 544.54	0.150
Calcium (mg)	759.98 ± 1.30	757.93 ± 1.28	0.908
Selenium (mg)	0.075 ± 1.36	0.069 ± 1.35	0.271
Folate (mcg)	255.85 ± 1.40	241.26 ± 1.44	0.649
Vitamin E (mg)	19.57 ± 1.71	17.58 ± 1.95	0.519
Vitamin C (mg)	125.62 ± 1.75	97.06 ± 1.72	0.052
β-carotene (mcg)	580.79 ± 2.68	443.36 ± 2.19	0.118
α-tocopherol (mg)	7.75 ± 1.78	6.71 ± 1.65	0.279
Dietary fiber (g)	18.13 ± 1.40	16.10 ± 1.57	0.506
Turmeric powder (teaspoon/day)	2.13 ± 0.81	2.36 ± 0.85	0.284

^1^Data are logarithmic-transformed mean ± standard deviation, except for energy, carbohydrate, fat, polyunsaturated fat, monounsaturated fat, potassium and turmeric, which are presented as mean ± standard deviation; ^2^P-values are from the independent-sample t test.

The crude within and between comparisons of anthropometric and biochemical values between the curcumin and placebo group are illustrated in Table 3. According to the results available, there was a significant reduction in anthropometric measurements among the adolescents in the curcumin group. In addition, a desirable improvement in lipid profile was detected in the intervention group. The results from the paired-sample t test showed that the participants in the placebo group only had significant reductions in weight and body mass index. There were no differences in the baseline and the end values for the variables between the two groups.

**Table 3.** The effect of curcumin supplementation on anthropometric and cardiovascular risk factors after 10 weeks of intervention^1^

Variable	Curcumin (n = 30)		Placebo (n = 30)		Within group		Between groups	
	Before	After	Before	After	P-value^2^	P-value^3^	P-value^4^	P-value^5^
Weight (kg)	83.55 ± 1.21	82.87 ± 1.21	81.52 ± 1.18	80.79 ± 1.17	0.060	0.042	0.599	0.581
BMI (kg/m^2^)	31.43 ± 2.84	31.00 ± 2.85	30.27 ± 2.83	30.00 ± 2.82	0.019	0.046	0.318	0.380
WC (cm)	100.31 ± 1.14	97.86 ± 1.13	97.07 ± 1.08	96.96 ± 1.09	0.008	0.918	0.245	0.738
HC (cm)	114.18 ± 2.77	113.17 ± 2.77	111.16 ± 2.76	110.53 ± 2.75	0.030	0.316	0.190	0.233
WHR	0.88 ± 1.06	0.86 ± 1.07	0.87 ± 1.07	0.87 ± 1.06	0.071	0.684	0.791	0.371
SBP (mmHg)	111.66 ± 7.85	111.36 ± 12.01	106.80 ± 11.66	109.56 ± 9.79	0.866	0.116	0.063	0.528
DBP (mmHg)	75.83 ± 9.05	75.14 ± 9.09	72.83 ± 7.62	73.72 ± 7.27	0.660	0.571	0.170	0.505
FBS (mg/dl)	87.23 ± 7.04	85.97 ± 7.02	86.23 ± 6.00	86.91 ± 4.88	0.205	0.335	0.556	0.546
Insulin (U/ml)	12.29 ± 1.71	14.81 ± 1.51	12.58 ± 1.55	13.58 ± 1.52	0.009	0.298	0.852	0.428
HOMA-IR	3.11 ± 1.93	3.41 ± 1.49	2.93 ± 1.19	3.17 ± 1.32	0.158	0.283	0.670	0.505
T-chol (mg/dl)	159.80 ± 27.72	161.41 ± 30.99	164.70 ± 27.78	162.32 ± 28.02	0.453	0.238	0.497	0.905
HDL (mg/dl)	48.93 ± 1.17	50.77 ± 1.18	50.96 ± 1.17	50.73 ± 1.20	0.042	0.826	0.325	0.985
LDL (mg/dl)	83.56 ± 1.33	85.12 ± 1.32	86.73 ± 1.27	85.41 ± 1.28	0.444	0.206	0.588	0.960
TG (mg/dl)	109.57 ± 1.48	102.16 ± 1.46	107.21 ± 1.57	106.30 ± 1.42	0.068	0.847	0.843	0.675
TG/HDL	2.49 ± 1.16	2.19 ± 0.95	2.39 ± 1.27	2.30 ± 0.98	0.021	0.499	0.765	0.659

BMI = body mass index; WC = waist circumference; HC = hip circumference; WHR = waist-to-hip ratio; SBP = systolic blood pressure; DBP = diastolic blood pressure; FBS = fasting blood sugar; HOMA-IR = homeostasis model assessment of insulin resistance; T-chol = total cholesterol; HDL = high-density lipoprotein; LDL = low-density lipoprotein; TG = triglycerides.

^1^Data are log-transformed mean ± standard deviation, except for SBP, DBP, FBS, HOMA-IR, TC and TG/HDL ratio, which are presented as mean ± standard deviation; ^2^within curcumin group, obtained from paired t test; ^3^within placebo group, obtained from paired t test; ^4^comparison of baseline values between curcumin and placebo groups using independent-sample t test; ^5^comparison of endpoint values between curcumin and placebo groups using independent-sample t test.

Table 4 presents the comparison of differences in the mean changes to anthropometric and biochemical indices after 10 weeks of supplementation. Regarding the results obtained, there were no significant differences between the two groups in the crude or adjusted models.

**Table 4.** Comparison of differences in means changes in anthropometric and biochemical indices among female adolescents between the curcumin and placebo groups^1^

Variable	Curcumin (n = 30)	Placebo (n = 30)	P-value^2^	P-value^3^
Weight (kg)	-0.71 ± 2.16	-0.81 ± 1.96	0.860	0.965
BMI (kg/m^2^)	-0.42 ± 0.91	-0.30 ± 0.74	0.567	0.426
WC (cm)	-2.54 ± 4.89	-0.05 ± 5.60	0.073	0.070
HC (cm)	-1.11 ± 2.68	-0.69 ± 3.26	0.592	0.833
WHR	-0.01 ± 0.03	0.003 ± 0.05	0.194	0.115
SBP (mmHg)	-0.30 ± 9.63	2.76 ± 9.36	0.216	0.418
DBP (mmHg)	-0.68 ± 8.44	0.88 ± 8.48	0.474	0.796
FBS (mg/dl)	-1.26 ± 5.33	0.68 ± 3.83	0.109	0.126
Insulin (U/ml)	1.94 ± 5.06	1.05 ± 5.28	0.506	0.619
HOMA-IR	0.30 ± 1.14	0.23 ± 1.17	0.823	0.909
T-chol (mg/dl)	1.61 ± 11.61	-2.37 ± 10.78	0.174	0.059
HDL (mg/dl)	1.91 ± 4.91	-0.04 ± 5.99	0.170	0.062
LDL (mg/dl)	1.57 ± 10.73	-1.24 ± 5.71	0.211	0.166
TG (mg/dl)	-8.34 ± 25.28	-5.57 ± 31.62	0.710	0.668
TG/HDL	-0.29 ± 0.66	-0.08 ± 0.70	0.246	0.191

BMI = body mass index; WC = waist circumference; HC = hip circumference; WHR = waist to hip ratio; SBP = systolic blood pressure; DBP = diastolic blood pressure; FBS = fasting blood sugar; HOMA-IR = homeostasis model assessment of insulin resistance; T-chol = total cholesterol; HDL = high-density lipoprotein; LDL = low-density lipoprotein; TG = triglycerides.

^1^Data are mean ± standard deviation; ^2^obtained from independent-sample t test; ^3^obtained from univariate analysis of covariance (ANCOVA) adjusted for turmeric intake, vitamin C, vitamin E, selenium, β-carotene intake and physical activity.

## DISCUSSION

The results from this parallel randomized trial study showed that curcumin supplementation for overweight and obese female adolescents over a 10-week period had a reducing effect on body mass index, waist circumference, hip circumference, high-density lipoprotein levels and triglyceride/high-density lipoprotein ratio and an increasing effect on insulin levels, in within-group analyses. Participants in the placebo group had significant reduction in weight and body mass index. Although we could not detect any significant differences between the intervention and placebo groups, the within-group results were considerable. This was the first time that the effects of curcumin supplementation had been studied among post-pubescent overweight or obese female adolescents in a developing country.

Previous studies have shown that childhood obesity and central obesity can be strong predictors for the presence of obesity and cardiovascular risk factors later in life.^22^^,^^23^ However, body mass index presents some limitations as a marker for obesity. It does not show individuals’ fat distribution or their degree of muscularity.^24^ It seems that measurements of waist circumference and central obesity are better than body mass index as indicators for cardiovascular risk factors among children and adolescents.^25^

In the present study, although we could not detect any significant difference between the intervention and placebo groups, curcumin supplementation significantly reduced body mass index, waist circumference and hip circumference in the intervention group after 10 weeks. Moreover, a trend towards significant reduction was detected in relation to body weight and waist-hip ratio (WHR) among the participants in the curcumin group. Participants in the placebo group had a significant reduction in weight and body mass index. The reduction in weight and body mass index in both groups can be attributed to prescribed diets. However, participants in the curcumin group had a significant reduction in their body size (i.e. waist circumference and hip circumference). The results from some previous studies are in line with those of the present study.^12^^,^^26^

It has been proposed that curcumin supplementation reduces body weight and body mass index by increasing the underlying metabolic rate,^27^ with downregulation of adipocytic transcriptional factors such as peroxisome proliferator-activated receptor γ and, therefore, suppression of preadipocyte differentiation.^27^^,^^28^ The results from one meta-analysis showed that curcumin supplementation had the capacity to reduce waist circumference, as seen in studies on subjects with body mass index less than 30 kg/m^2^ and studies with durations of more than eight weeks.^29^ However, further studies are needed to determine the potential mechanism of curcumin for reducing abdominal obesity and visceral fat.

In this study, we documented a significant increase in insulin levels after 10 weeks of curcumin supplementation. However, fasting blood sugar levels and the homeostasis model assessment of insulin resistance did not change significantly. A previous study was unable to show any beneficial effect from a hypocaloric diet on fasting insulin and blood glucose levels in obese children, even after reducing the calorie intake required for weight maintenance by 30%.^29^ Therefore, it seems that this significant increase in insulin levels is mediated through curcumin intake.

Some studies have shown that curcumin supplementation is effective in reducing fasting glucose levels and insulin resistance in diabetic patients.^8^^,^^30^ However, other surveys on non-diabetic patients failed to prove that curcumin produced any significant reducing effect on glycemic indices.^11^^,^^31^ In the current study, a significant increase in insulin levels was observed among the participants in the curcumin group.

Curcumin consumption may increase insulin secretion from pancreatic cells and improve pancreatic function over the course of time.^32^ The results from a study on animals suggested that curcumin intake might increase insulin secretion by increasing stimulation of glucagon-like peptide-1 secretion.^33^ The results from an *in-vitro* study also confirmed the insulin-releasing effect and stimulating action of turmeric in cell cultures from the pancreas and muscle tissues of adult mice.^34^ However, for more precise interpretation of such results, larger clinical trials with higher doses of curcumin are needed.

In the present study, there were significant improvements in high-density lipoprotein levels and the triglyceride/high-density lipoprotein ratio among the adolescents in the curcumin group. In addition, a marginally significant reduction in triglyceride levels was observed. When differences in mean change were compared, a marginally significant effect was detected in relation to total cholesterol and low-density lipoprotein levels. In this context, the results from previous studies are similar to those of the current study.^12^^,^^26^

It has been shown that the prevalence of some cardiovascular risk factors in Middle Eastern countries are completely different from those in other parts of the world (i.e. low levels of high-density lipoprotein cholesterol and high levels of triglycerides).^35^ Documenting this result among Iranian people is of great importance, given that consumption of refined carbohydrate is more prevalent among this population and since one of the obvious features of this population is the presence of a hypertriglyceridemic waist phenotype.^36^

The results from the present study also showed that curcumin intake could improve this specific dyslipidemia (i.e. low levels of high-density lipoprotein cholesterol and high levels of triglycerides) among adolescents, without the need for any significant restriction of carbohydrate consumption. A previous study showed that curcumin supplementation might increase total cholesterol slightly.^10^ Administration of curcumin can improve the lipid profile through increasing fatty acid β-oxidation and lipoprotein lipase, suppressing fatty acid synthase and downregulating lipogenic genes and enzymes such as sterol-regulatory element-binding protein-1, acetyl-CoA carboxylase and peroxisome proliferator-activated receptor-α.^11^^,^^37^ In addition, through increasing the expression of ABCG1 (ATP-binding cassette subfamily G member 1), curcumin may increase serum high-density lipoprotein levels by enhancing high-density lipoprotein-dependent lipid efflux.^38^

To the best of our knowledge, this was the first time that the effects of curcumin supplementation in parallel with a diet leading to slight weight loss were assessed in relation to cardiometabolic risk factors among post-pubescent overweight or obese female adolescents in a developing country. The participants received similar diets for slight weight loss with specific macronutrient distribution, as a basic intervention to control for the confounding effect of dietary intakes. Moreover, the effect of potential confounders was taken into consideration in the statistical analysis. In the current study, we included otherwise healthy overweight and obese girls. Therefore, it would be possible to generalize the results from this study to similar populations of adolescents.

However, several limitations might affect the final results. Due to financial limitations, we were unable to determine the serum levels of curcumin in order to confirm the participants’ adherence. Therefore, their compliance with the prescribed supplements was checked through other possible ways. Furthermore, for better interpretation of the findings, particularly glycemic indices and insulin resistance status, we would have needed to assess the serum free fatty acid levels. However, we were unable to do this due to budget limitations. Although the number of withdrawals among the subjects during the study period was negligible, the missing data from these subjects could have affected the final results from this study. However, we tried to reduce this effect by predicting the missing values through linear regression analysis.

## CONCLUSIONS

Prescription of curcumin supplementation along with a diet leading to mild weight loss may have beneficial effects on some cardiovascular risk factors in overweight and obese female adolescents. Larger clinical trials with higher doses of curcumin and longer duration are needed to confirm the results from the current study.

## References

[1] Katzmarzyk PT, Barreira TV, Broyles ST (2015). Relationship between lifestyle behaviors and obesity in children ages 9-11: Results from a 12-country study. Obesity (Silver Spring).

[2] Sommer A, Twig G (2018). The Impact of Childhood and Adolescent Obesity on Cardiovascular Risk in Adulthood: a Systematic Review. Curr Diab Rep.

[3] Jafari-Adli S, Jouyandeh Z, Qorbani M (2014). Prevalence of obesity and overweight in adults and children in Iran; a systematic review. J Diabetes Metab Disord.

[4] Umer A, Kelley GA, Cottrell LE (2017). Childhood obesity and adult cardiovascular disease risk factors: a systematic review with meta-analysis. BMC Public Health.

[5] Magriplis E, Farajian P, Panagiotakos DB, Risvas G, Zampelas A (2018). The relationship between behavioral factors, weight status and a dietary pattern in primary school aged children: The GRECO study. Clin Nutr.

[6] Fernández-Barrés S, Romaguera D, Valvi D (2016). Mediterranean dietary pattern in pregnant women and offspring risk of overweight and abdominal obesity in early childhood: the INMA birth cohort study. Pediatr Obes.

[7] Shehzad A, Ha T, Subhan F, Lee YS (2011). New mechanisms and the anti-inflammatory role of curcumin in obesity and obesity-related metabolic diseases. Eur J Nutr.

[8] Chuengsamarn S, Rattanamongkolgul S, Luechapudiporn R, Phisalaphong C, Jirawatnotai S (2012). Curcumin extract for prevention of type 2 diabetes. Diabetes Care.

[9] Sahebkar A (2014). A systematic review and meta-analysis of randomized controlled trials investigating the effects of curcumin on blood lipid levels. Clin Nutr.

[10] Baum L, Cheung SK, Mok VC (2007). Curcumin effects on blood lipid profile in a 6-month human study. Pharmacol Res.

[11] Panahi Y, Kianpour P, Mohtashami R (2016). Curcumin Lowers Serum Lipids and Uric Acid in Subjects With Nonalcoholic Fatty Liver Disease: A Randomized Controlled Trial. J Cardiovasc Pharmacol.

[12] Rahimi HR, Mohammadpour AH, Dastani M (2016). The effect of nano-curcumin on HbA1c, fasting blood glucose, and lipid profile in diabetic subjects: a randomized clinical trial. Avicenna J Phytomed.

[13] World Health Organization (2007). BMI-for-age GIRLS.

[14] Kelishadi R, Ardalan G, Gheiratmand R (2007). Association of physical activity and dietary behaviours in relation to the body mass index in a national sample of Iranian children and adolescents: CASPIAN Study. Bull World Health Organ.

[15] Rouhani MH, Kelishadi R, Hashemipour M, Esmaillzadeh A, Azadbakht L (2013). The effect of low glycemic index diet on body weight status and blood pressure in overweight adolescent girls: a randomized clinical trial. Nutr Res Pract.

[16] Mahan LK, Raymond JL, Lysen LK, Israel DA (2017). Nutrition in Weight Management. Krause’s Food & the Nutrition Care Process.

[17] (2011). Healthy Eating Plate dishes out sound diet advice. More specific than MyPlate, it pinpoints the healthiest food choices. Harv Heart Lett.

[18] Azadbakht L, Mirmiran P, Hosseini F, Azizi F (2005). Diet quality status of most Tehranian adults needs improvement. Asia Pac J Clin Nutr.

[19] Matthews DR, Hosker JP, Rudenski AS (1985). Homeostasis model assessment: insulin resistance and beta-cell function from fasting plasma glucose and insulin concentrations in man. Diabetologia.

[20] Saraf-Bank S, Ahmadi A, Paknahad Z, Maracy M, Nourian M (2019). Effects of curcumin supplementation on markers of inflammation and oxidative stress among healthy overweight and obese girl adolescents: A randomized placebo‐controlled clinical trial. Phytother Res.

[21] Hashemipour M, Kelishadi R, Shapouri J (2009). Effect of zinc supplementation on insulin resistance and components of the metabolic syndrome in prepubertal obese children. Hormones (Athens).

[22] Dekkers JC, Podolsky RH, Treiber FA (2004). Development of general and central obesity from childhood into early adulthood in African American and European American males and females with a family history of cardiovascular disease. Am J Clin Nutr.

[23] Lawlor DA, Benfield L, Logue J (2010). Association between general and central adiposity in childhood, and change in these, with cardiovascular risk factors in adolescence: prospective cohort study. BMJ.

[24] Heymsfield SB, Scherzer R, Pietrobelli A, Lewis CE, Grunfeld C (2009). Body mass index as a phenotypic expression of adiposity: quantitative contribution of muscularity in a population-based sample. Int J Obes (Lond).

[25] Jahagirdar R, Hemchand KP, Chiplonkar SA, Khadilkar VV, Khadilkar AV (2012). Relationship between body mass index, fat distribution and cardiometabolic risk factors in Indian children and adolescents. Pediatr Obes.

[26] Rahmani S, Asgary S, Askari G (2016). Treatment of Non-alcoholic Fatty Liver Disease with Curcumin: A Randomized Placebo-controlled Trial. Phytother Res.

[27] Ejaz A, Wu D, Kwan P, Meydani M (2009). Curcumin inhibits adipogenesis in 3T3-L1 adipocytes and angiogenesis and obesity in C57/BL mice. J Nutr.

[28] Zhao J, Sun XB, Ye F, Tian WX (2011). Suppression of fatty acid synthase, differentiation and lipid accumulation in adipocytes by curcumin. Mol Cell Biochem.

[29] Parillo M, Licenziati MR, Vacca M, De Marco D, Iannuzzi A (2012). Metabolic changes after a hypocaloric, low-glycemic-index diet in obese children. J Endocrinol Invest.

[30] Na LX, Li Y, Pan HZ (2013). Curcuminoids exert glucose-lowering effect in type 2 diabetes by decreasing serum free fatty acids: a double-blind, placebo-controlled trial. Mol Nutr Food Res.

[31] Tang M, Larson-Meyer DE, Liebman M (2008). Effect of cinnamon and turmeric on urinary oxalate excretion, plasma lipids, and plasma glucose in healthy subjects. Am J Clin Nutr.

[32] Ghorbani Z, Hekmatdoost A, Mirmiran P (2014). Anti-hyperglycemic and insulin sensitizer effects of turmeric and its principle constituent curcumin. Int J Endocrinol Metab.

[33] Kato M, Nishikawa S, Ikehata A (2017). Curcumin improves glucose tolerance via stimulation of glucagon-like peptide-1 secretion. Mol Nutr Food Res.

[34] Mohankumar S, McFarlane JR (2011). An aqueous extract of Curcuma longa (turmeric) rhizomes stimulates insulin release and mimics insulin action on tissues involved in glucose homeostasis in vitro. Phytother Res.

[35] Esmaillzadeh A, Azadbakht L (2008). Food intake patterns may explain the high prevalence of cardiovascular risk factors among Iranian women. J Nutr.

[36] Esmaillzadeh A, Mirmiran P, Azizi F (2005). Whole-grain intake and the prevalence of hypertriglyceridemic waist phenotype in Tehranian adults. Am J Clin Nutr.

[37] Sahebkar A (2014). Curcuminoids for the management of hypertriglyceridaemia. Nat Rev Cardiol.

[38] Peschel D, Koerting R, Nass N (2007). Curcumin induces changes in expression of genes involved in cholesterol homeostasis. J Nutr Biochem.

